# S142. RESTING STATE NETWORKS ALTERATION IN BIPOLAR DEPRESSION

**DOI:** 10.1093/schbul/sby018.929

**Published:** 2018-04-01

**Authors:** Gianluca Mingoia, Igor Nenadic

**Affiliations:** 1 Aachen University; 2 Jena University Hospital

## Abstract

**Background:**

While functional MRI and PET studies have shown altered task-related brain activity in bipolar depression, recent studies suggest that such differences might also be found in the resting state (RS). Here we used ICA based analysis to investigate RS fMRI data to compare connectivity of 11 well known networks (Auditory, Cerebellum, DMN, Exectutive Control, Fronto-parietal 1, Fronto-parietal 2, Salience, Sensorimotor, Visual1, Visual2, Visual3 network) between patients with bipolar depression and healthy controls suggesting deficits in related neuropsychological functions.

**Methods:**

We obtained RS fMRI series (3T, 3x3x3mm resolution, 45 slices, TR 2.55s, 210 volumes) in 22 bipolar patients (mean age 38.4a±11.3), on stable medication and 22 matched healthy controls (36.8a±11.7).

Subjects were asked to lie in the scanner keeping eyes closed with no further specific instructions. Data were pre-processed; we applied FSL MELODIC (pICA) yielding IC, we used FIX to auto-classify ICA components which represent artifacts and an automated routine to select for each subject the component matching the anatomical definition of resting state networks.

SPM12 was used for second level analysis, we used two sample t-test to compare networks functional connectivity between groups.

**Results:**

Our method reliably identified all networks in every controls and patients. We found significant differences in the anatomical pattern of areas. Patients showed decreased functional connectivity in comparison to healthy controls in portions Cerebellum, DMN, Fronto-parietal1, Fronto-parietal2, Visual1, Visual2 and Visual3 networks; in addition, patients showed increased functional connectivity in comparison to healthy controls in portions of Cerebellum Frontoparietal1 networks.

The power spectrum of the bipolar patients and healthy control time courses don’t differ significantly in any of the brain networks, but there is a slight difference between the average slope between bipolar and healthy subject, Total Av. Bip = -0.88743 and Total Av. HC = -0.90282.

**Discussion:**

Well-known resting state networks were reliable identified from RS fMRI in Bipolar depression patients. The differences in anatomical distribution point to possible alterations in functional connectivity in Bipolar depression, which suggests disruption in cerebellum, DMN, fronto-parietal and visual neuropsychological related activity.

